# Food Insecurity and Effectiveness of Behavioral Interventions to Reduce Blood Pressure, New York City, 2012–2013

**DOI:** 10.5888/pcd12.140368

**Published:** 2015-02-12

**Authors:** Stephanie A. Grilo, Amanda J. Shallcross, Gbenga Ogedegbe, Taiye Odedosu, Natalie Levy, Susan Lehrer, William Chaplin, Tanya M. Spruill

**Affiliations:** Author Affiliations: Stephanie A. Grilo, Yale School of Public Health, New Haven, Connecticut; Amanda J. Shallcross, Gbenga Ogedegbe, New York University School of Medicine, New York, New York; Taiye Odedosu, Natalie Levy, Bellevue Hospital Center, New York, New York; Susan Lehrer, Health and Home Care, Health and Hospitals Corporation, New York, New York; William Chaplin, St John’s University, Queens, New York.

## Abstract

**Introduction:**

Food insecurity is associated with diet-sensitive diseases and may be a barrier to successful chronic disease self-management. To evaluate the impact of food insecurity on blood pressure reduction in a pilot clinical trial, we tested the effectiveness of 2 behavioral interventions for hypertension in people with and without food security.

**Methods:**

A group of 28 men and women with type 2 diabetes and uncontrolled hypertension were randomized to either 1) home blood pressure telemonitoring alone or 2) home blood pressure telemonitoring plus telephone-based nurse case management. The primary outcome was 6-month change in systolic blood pressure.

**Results:**

The 2 interventions resulted in modest, nonsignificant blood pressure reductions. Food-secure patients experienced clinically and statistically significant reductions in blood pressure, whereas no significant change was seen among food-insecure patients.

**Conclusion:**

Screening for food insecurity may help identify patients in need of tailored disease management interventions.

## Introduction

Food insecurity affects 14.9% of American households, and rates are approaching 25% among black and Hispanic households (1). The US Department of Agriculture (USDA) definition of food insecurity is “having limited or uncertain availability of nutritionally adequate and safe foods or limited or uncertain ability to acquire acceptable foods in socially acceptable ways” (1). Nutritionally poor foods are often less expensive than healthful foods (2,3), and food insecurity is associated with poor diet quality (4–6) and diet-sensitive diseases, including diabetes, hypertension, and hyperlipidemia (7–12). Food insecurity has also been associated with other behavioral factors related to chronic disease self-management (10,11,13) and poor disease control (13–16). On the basis of these findings, we propose that food-insecure patients may be less likely to benefit from behavioral interventions designed to improve chronic disease outcomes, but to our knowledge this relationship has not been tested.

Our study examined the impact of food insecurity on blood pressure (BP) reduction in a pilot study examining the comparative effectiveness of 2 hypertension self-management strategies in an urban sample of low-income black and Hispanic patients. We tested the hypothesis that the interventions would be less effective in reducing BP over 6 months among patients who were food-insecure versus food-secure.

## Methods

### Participants and procedures

Participants were 28 English- or Spanish-speaking men and women with uncontrolled hypertension and comorbid type 2 diabetes. Patients were recruited from the Ambulatory Care Clinic at Bellevue Hospital in New York City via flyers, face-to-face recruitment, and physician referral. Inclusion criteria were as follows: 18 years of age or older, diagnosis of type 2 diabetes (confirmed in the electronic health record [EHR]), received care at the practice for at least 6 months, uncontrolled hypertension (defined as systolic blood pressure (SBP) 140 mm Hg or higher and/or diastolic blood pressure (DBP) 90 mm Hg or higher on at least 2 previous visits in the previous year and at study screening), and proficiency in either English or Spanish. Exclusion criteria were screening BP 180/110 mm Hg or higher, cognitive dysfunction or psychiatric comorbidity, pregnancy, concurrent participation in another clinical trial, or being deemed unable to comply with study protocol (ie, unwilling or unable to follow the home BP monitoring protocol or participate in telephone sessions).

Patients who were interested and eligible provided written informed consent and completed baseline assessments, including self-report questionnaires and measurement of height, weight, and BP. The average of 3 BP readings taken with a validated automated device (Watch BP Office, Microlife Medical Home Solutions, Inc) was recorded. Participants were randomly assigned to either 1) home BP telemonitoring (HBPTM) alone; or 2) home BP telemonitoring plus nurse case management (HBPTM+NCM). Participants assigned to either group completed follow-up assessments at 3 and 6 months, at which time BP measurement was repeated. Participants received $10 for completion of the 3-month visit and $15 for completion of the 6-month visit.

### Study interventions

#### Home BP telemonitoring (HBPTM)

Participants received a validated home BP monitoring device (Stabil-O-Graph mobil) and were trained by a research assistant in its use. They were instructed to take BP readings in the morning and evening at least 3 days per week during the 6-month intervention. Readings were wirelessly transmitted to a secure central server, and reports were sent to patients’ physicians via secure email before scheduled appointments. For safety measures, the monitors were preprogrammed with BP alarm values, which, when triggered, activated an email to the research staff or nurse case manager, depending on the participant’s study group, prompting follow-up with the patient. Participants assigned to home telemonitoring received publicly available educational materials regarding management of hypertension and diabetes and were encouraged to follow the clinical guidelines described in the materials. They also completed a single 30-minute telephone session with a nurse case manager within 2 weeks of randomization and delivery of the blood pressure telemonitoring device to their home.

#### Home BP telemonitoring plus nurse case management (NCM+HBPTM)

The combined intervention supplements the home blood pressure telemonitoring protocol with patient self-management support from a nurse case manager. The intervention tested in this study was the HouseCalls telehealth program, which is integrated into the New York City Health and Hospitals Corporation (HHC) system as part of its home care program. The intervention is delivered by HHC nurses who have real-time access to patients’ EHRs and are in communication with their providers.

Within 2 weeks of randomization and delivery of the home telemonitoring device, a nurse case manager contacted patients to make sure they were comfortable using the device and to initiate the planned schedule of counseling telephone calls: weekly for months 1 and 2, biweekly for month 3, and monthly for months 4 through 6. The nurse case manager had access to the patients’ home BP data via a secure website, where the readings are displayed in easy-to-read charts and figures that highlight the control rate for each week. This information was used by the nurse case manager as a basis for counseling sessions with the patient. The patient’s physician(s) received home BP reports via secure email before every scheduled appointment for the duration of the study.

During scheduled telephone sessions, HouseCalls nurse case managers provide self-management education and medication and appointment reminders, and they facilitate patient–provider communication. They also create individually tailored goals in collaboration with each participant. Target behaviors may include dietary changes, physical activity, weight loss, smoking cessation, stress reduction, self-monitoring of blood glucose and BP, and medication adherence. The nurse case managers assess patients’ barriers to behavior change and use problem-solving and motivational interviewing techniques to support behavior change efforts. At the end of each counseling session, the nurse case managers record the notes of each encounter in the patient’s EHR and communicate with the patient’s physician if needed (eg, regarding medication side effects or the need for appointments or medication refills). Calls are 15 to 45 minutes long depending on the needs of the patient.

### Assessment of food insecurity

Participants completed the 6-item USDA food security short form at baseline (17,18). Sample items are “The food that (I/we) bought just didn’t last, and (I/we) didn’t have money to get more”; (“I/we) couldn’t afford to eat balanced meals”; and “Were you ever hungry but didn’t eat because there wasn’t enough money for food?” Items were rated for the previous 12 months using several response scales. Responses of “often” or “sometimes”; “yes”; and “almost every month” or “some months but not every month” were coded as affirmative, and the sum of affirmative responses yielded the scale score (0–6). A score of 0 or 1 indicates high or marginal food security, 2 to 4 indicates low food security, and 5 to 6 indicates very low food security. We used a score of 2 or more to define food insecurity (18).

### Statistical analysis

Because the primary hypothesis was tested in the context of a randomized trial, linear multilevel repeated-measures regression analyses were first performed to generate estimates of SBP change from baseline to 6 months between intervention arms (Group × Time interaction). All participants were included in this intent-to-treat analysis, and missing data were handled by using full-information maximum likelihood estimates. Following this analysis, we tested the hypothesis that food insecurity would be associated with smaller reductions in BP (Food Insecurity × Time interaction). After performing an unadjusted analysis, we also examined the association between food insecurity and SBP change after adjusting for a set of relevant covariates (age, sex, race/ethnicity, total family income adjusted for household size, education, body mass index, and antihypertensive medication use). Analyses were performed with SPSS version 20 (IBM Corp).

## Results

Sample characteristics show that this sample was racially/ethnically diverse, with low socioeconomic position (Table 1). This result was expected, given the patient population served by Bellevue Hospital. Hispanic participants were more likely than black participants to be food insecure (*P* = .04); no other demographic or baseline characteristics were related to food security status. Of the 28 enrolled participants, 23 (82%) completed the 6-month visit. The primary reasons for dropout were family or housing issues and leaving the area. Dropout was not associated with intervention arm, food security status, or baseline BP (*P* values > .80). Regarding adherence to the interventions, no significant differences were seen between food-secure and food-insecure participants in the number of home BP readings transmitted (17.8 vs 18.8, *P* = .80) or in the number of telephone sessions completed among those in the HBPTM+NCM group (8.2 vs 9.3, *P* = .68).

**Table 1:** Demographic and Baseline Characteristics of a Sample of Patients With Type 2 Diabetes and Uncontrolled Hypertension (N = 28), New York City, 2012–2013

Participant Characteristic	Value
**Age, y, mean (SD)**	60.7 (8.2)
**Sex, % female**	57.1
**Race/ethnicity, %**	
Hispanic	71.4
Black	28.6
**Annual income (adjusted for household size), $, mean (SD) **	7,044 (6,271)
**Education, %**	
Less than high school graduate	35.6
High school graduate or GED	35.7
Some college	17.9
Bachelor’s or associate’s degree	10.7
**Food insecure, %**	57.1
**Body mass index, kg/m^2^, mean (SD)**	33.8 (6.7)
**Use antihypertensive medications, %**	96.4
**Systolic BP, mm Hg, mean (SD)**	154.8 (10.4)
**Diastolic BP, mm Hg, mean (SD)**	85.7 (9.3)

Abbreviations: BP, blood pressure; GED, general educational development certificate; SD, standard deviation.

The intent-to-treat analysis showed a nonsignificant reduction in SBP of 2.7 mm Hg from baseline to 6 months across both intervention arms (main effect of Time). The Group × Time interaction was not significant, indicating no difference in the efficacy of the 2 interventions. The analysis of the primary hypothesis indicated a significant Food Insecurity × Time interaction. Simple slope analyses (19) showed that the interventions significantly decreased SBP among food-secure participants (b = −0.77, *t* = −4.35, *P* < .001) but had no significant impact on SBP among food-insecure participants (b = 0.25, *t* = 1.52, *P* = .14). Overall, the estimated drop in SBP over the course of the intervention (adjusting for the set of covariates) among food-secure participants was 9.2 mm Hg, whereas SBP increased by 3.1 mm Hg among food-insecure participants (Figure). In each of the analyses, results were not substantially different in magnitude or significance after adjustment for intervention arm, age, sex, race/ethnicity, family income, education, body mass index, and antihypertensive medication use (Table 2).

**Figure:**
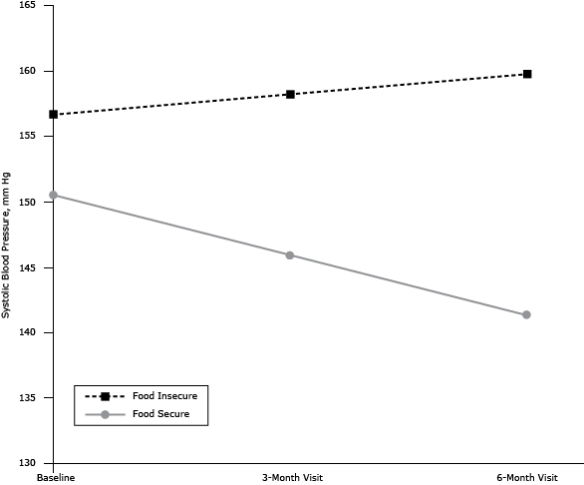
Food Insecurity × Time interaction effect on systolic blood pressure (SBP) among 28 patients with uncontrolled hypertension and diabetes receiving telemonitoring interventions for BP reduction, New York City, 2012–2013. Values depict unadjusted SBP estimates at each time point.

**Table 2:** Unstandardized Regression Coefficients Representing Effects on Systolic Blood Pressure (mm Hg/week)^a^

Variable	Unadjusted			Adjusted		
	b	*t*	*P* Value	b	*t*	*P* Value
**Model 1**: Main effect of Time	−.23	−1.42	.17	−.15	−.96	.35
**Model 2:** Main effect of Food Insecurity status	−3.11	−.78	.45	7.34	1.01	.33
**Model 3:** Intervention × Time	−.004	−.013	.99	-.09	−.25	.80
**Model 4:** Food Insecurity Status × Time	1.02	4.22	.001	1.01	3.88	.001
Effect of Time	−.77	−4.35	<.001	−.66	−.35	.002
Effect of Food Insecurity status	−2.45	−.61	.55	−.22	−.30	.77

a Adjusted models control for intervention arm, age, sex, race/ethnicity, total family income adjusted for household size, education, body mass index, and antihypertensive medication use.

## Discussion

The 2 hypertension self-management interventions resulted in modest, nonsignificant BP reductions in this sample of urban, minority, low-income patients. There was no significant difference between the interventions. As hypothesized, food security status was a moderator of intervention effects: food-secure patients experienced clinically and statistically significant reductions in BP while there was no significant change among food-insecure patients. The results held when adjusting for income, suggesting that food insecurity is associated with negative outcomes above and beyond this related risk factor.

These findings contribute to the growing literature demonstrating poorer health outcomes in food-insecure versus food-secure individuals (7–14). Results of this study suggest that hypertension self-management interventions based on traditional behavioral recommendations are unlikely to improve BP in food-insecure patients. The negative impact of food insecurity may reflect limitations on these patients’ ability to follow dietary recommendations (4–6), which were included in both interventions. Racial/ethnic minority patients experiencing food insecurity may be at particularly high risk for negative outcomes given poorer health literacy and cultural beliefs that may further influence negative food choices. However, detailed dietary data were not collected in this pilot study, so these hypotheses could not be tested.

We did conduct exploratory analyses of several potential factors that might help to explain the observed differences in BP change over time between patients with and without food security. We found no significant differences in self-reported medication adherence based on the 8-item Morisky Medication Adherence Scale (20) or health insurance coverage, though we did not have data specifically regarding prescription drug coverage. We also did not collect data on health literacy (21), a potentially relevant factor in this patient sample, or on psychological stress, which affects BP and is part of a proposed model of the cycle of food insecurity and chronic disease (9,22–24).

In addition to the lack of data concerning possible explanatory factors, the small sample size is a limitation that precludes generalizations. Another limitation is the 1-time assessment of food insecurity, given that food security status varies over time, both within a month and over longer periods of time (25). The duration and pattern of exposure to food insecurity may be a predictor of the associated health consequences. We hope in the future to build on the findings from this pilot study by conducting more detailed analyses of the effects of food insecurity, which will inform the need for tailoring interventions in terms of approach and content.

Although preliminary, results of this pilot study suggest that food insecurity may be a novel intervention target for patients with hypertension and other diet-sensitive chronic diseases. In 2012, 59% of food-insecure households participated in at least 1 of the 3 largest federal food and nutrition assistance programs (1). Increasing use of available resources may be a useful approach to helping food-insecure patients overcome barriers to adopting healthful behaviors. The development of innovative, tailored strategies to help food-insecure people follow dietary recommendations is an area for future research that will improve disease self-management efforts and health outcomes in this vulnerable population.
